# Niobium Nitride Preparation for Superconducting Single-Photon Detectors

**DOI:** 10.3390/molecules28176200

**Published:** 2023-08-23

**Authors:** Peng Luo, Yihui Zhao

**Affiliations:** 1 School of Optics and Photonics, Beijing Institute of Technology, Beijing 100081, China; 2 Key Laboratory of Photoelectronic Imaging Technology and System, Ministry of Education of the People’s Republic of China, Beijing 100081, China

**Keywords:** single-photon detectors, deposition processes, superconductor, nanowire

## Abstract

Niobium nitride (NbN) is widely used in the production of superconducting nanowire single-photon detectors (SNSPDs) due to its high superconducting transition temperature and suitable energy gap. The processing parameters used for the preparation of NbN films and the subsequent processing of nanowires have a significant effect on the performance of the SNSPD. In this review, we will present various thin film growth methods, including magnetron sputtering, atomic layer deposition (ALD), and chemical vapor deposition (CVD). The relationships between the superconducting performance of each thin film and the corresponding deposition process will be discussed. Subsequently, NbN nanowire fabrication methods and microstructures based on thin film etching will be summarized, and their impact on the qualities of the finished SNSPDs will be systematically analyzed. Finally, we will provide an outlook for the future development of preparation for SNSPD.

## 1. Introduction

Superconducting nanowire single-photon detectors (SNSPDs), which work in the superconducting critical state, have shown great potential for ultra-sensitive detection, such as quantum key communication [1,2], laser ranging [3], and biological detection [4,5]. Since it was developed by Gol’tsman et al. in 2001 [6], it has been demonstrated that the SNSPD has great advantages—including a 100% theoretical detection efficiency, low dark counting rate (10^−4^ counts per second (ps) [7]), and low timing jitter (4.6 ps [8])—compared with other single-photon detectors, such as the avalanche photodiode (APD) and the photomultiplier tube (PMT).

The development of superconducting materials, which are used to make the most important components of SNSPDs, has been the subject of considerable research efforts. Polycrystalline Nb-based superconducting materials such as NbN [7], as well as amorphous materials such as WSi [9,10] and MoSi [11,12], have been widely used in the fabrication of SNSPDs. Among all the reported materials, NbN has shown excellent superconducting properties (i.e., ~16 K theoretical superconducting transition temperature, a penetration depth greater than 180 nm, and ~5 nm short coherence length) and outstanding chemical and mechanical stability. To date, system detection efficiencies of over 90% have been reported for NbN-based SNSPDs [7,13,14].

In the production of SNSPDs, there are two steps: the deposition of a superconducting thin film and, subsequently, the fabrication of nanowires from the deposited film. In practice, the parameters for these two processes must be well-optimized to meet strict requirements. The capacity of the SNSPD depends on the quality of the superconducting thin film, i.e., the film should have a compact and uniform structure, precisely controlled physical and chemical defect densities, and an accurate thickness. For example, accurate control of the deposition thickness of the superconducting NbN layer is required because the thickness is more important here than in other applications—such as the fabrication of Josephson junctions—for which NbN films are commonly used [15,16]. In addition, inconsistencies in the etched nanowire are considered one of the main causes of detector dark count and timing jitter [17]. As a consequence, process selection and parameter control are crucial aspects of both the thin film deposition and nanowire etching processes involved in the production of SNSPDs. Therefore, it is necessary to summarize the relationship between detector performance and the preparation approach used in these two core processes, i.e., the deposition of the superconducting thin film and the subsequent etching of the nanowires.

In this review, we introduce various NbN thin film deposition and processing methods as well as optimization techniques. We also demonstrate the relationships between the quality of the thin films produced using the different preparation parameters and the performance of the resulting SNSPD. The techniques and processes used to etch NbN nanowires from the deposited NbN thin films are subsequently summarized. We provide overviews of the quality of the nanowires produced using the various fabrication methods, e.g., their consistency and uniformity, as well as their impact on SNSPD performance. Furthermore, the different geometric patterns of the etched superconducting nanowires and their effects on SNSPD performance are reviewed.

## 2. NbN Thin Film Preparation

NbN thin film preparation is the first step in the fabrication of an SNSPD. The preparation of the thin film includes the choice of substrate, the deposition technique, and the design of the process parameters. Here, we focus on different deposition techniques for NbN thin films, including magnetron sputtering, atomic layer deposition (ALD), and other reported deposition methods. In addition, the techniques used in the preparation of NbTiN superconducting thin films are reviewed and analyzed.

### 2.1. Magnetron Sputtering Deposition

Magnetron sputtering is a physical vapor deposition (PVD) technique, and it is reportedly the most widely used method for preparing NbN thin films [6,18,19,20]. In the mature magnetron sputtering deposition process, it has been found that various process conditions, such as the substrate material, the substrate temperature, the nitrogen partial pressure (nitrogen content), the radio frequency bias, and the deposition thickness, can affect the structure and superconducting properties of the films. Ultimately, these may lead to significant changes and impact the various aspects of SNSPD performance, e.g., detection sensitivity, detection efficiency, spectral response range, dark count, and timing jitter.

**Figure 1:**
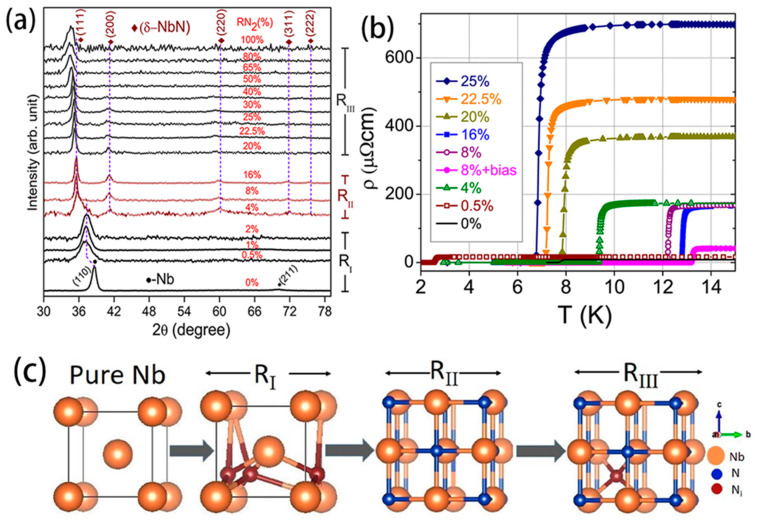
The effect of nitrogen partial pressure and disorder on film structure and superconducting transition temperature. (**a**) X-ray Diffraction (XRD) patterns of Nb-N films under different N_2_ partial pressures; R_II_ exhibits good NbN stoichiometric phase. Copyright 2021, Elsevier. (**b**) The change in film resistivity with temperature under different nitrogen partial pressures. The highest $T_{C}$ is observed at 8% nitrogen partial pressure and with the addition of radiofrequency bias. Copyright 2021, Elsevier. (**c**) A schematic diagram mimicking the evolution of Nb-N phases with increasing R_N2_. Here, large circles represent Nb atoms, and smaller ones represent N atoms. Copyright 2021, Elsevier.

In earlier work, researchers mainly used magnetron sputtering to prepare NbN, and they changed the sputtering parameters to study the superconducting properties of thin films. Alessandrini et al. [18] summarized the relationships between the properties of niobium nitride thin films and the basic operating parameters of radio frequency magnetron sputtering. An NbN_x_ (the ratio of N to Nb content in NbN materials is defined as X) with a stoichiometry of x = 0.91 exhibits excellent superconducting properties and the highest theoretical superconducting transition temperature (16.1 K). A considerable body of experimental evidence has substantiated the influence of the crystal structure of Nb nitrides on their superconducting transition temperature. Table 1 shows various NbN crystal structures and their corresponding reference transition temperatures. Kalal et al. [21] studied the effect of nitrogen partial pressure (R_N2_$\left[R_{N2}=P_{N2}/(P_{N2}+P_{Ar})\right]$) on the process of producing nitride films. As is shown in Figure 1, the R_I_ region of a thin film is formed with interstitial incorporation of N atoms within the bcc Nb. Interestingly, even a small amount of N atoms is sufficient to cause the XRD peak position to shift from 38.6° (pure Nb) to 37.2°. Unlike pure Nb, the crystal structure undergoes lattice parameter (LP) expansion and a reduction in crystallite size. When R_N2_ = 8% and 16% in region R_II_, they belong to the δ-NbN structure and have LP values of 4.368 and 4.376, respectively. Experimental results have shown that thin films in this region have higher superconducting transition temperatures. In region R_III_, there is an excess of N atoms in NbN; the (111) peak shifts towards the lower 2θ and broadens, indicating lattice expansion and distortion in the NbN. The schematic diagram illustrating the evolution of Nb-N phases depicts the mechanism by which N_2_ gas forms the Nb-N phase structure. Alfonso et al. [22] investigated the influence of certain fabrication parameters, including substrate temperature, sputtering power, and nitrogen flux, on the crystallization and microstructures of NbN films. Their results showed that with a constant power of 300 W and a nitrogen flux, increasing the substrate temperature causes the NbN film to grow along the (111) plane of the δ-NbN phase structure, and with the same power and a 553 K substrate temperature, an increase in the nitrogen flux favors the growth of the NbN film along the (200) plane [22].

It is evident that the thickness of NbN films also affects their superconducting properties [23,24,25,26]. Kang et al. [26] grew epitaxial films with thicknesses ranging from 2.5 to 100 nm on MgO substrate and compared the superconducting properties. The superconducting transition temperature has a significant dependence on thickness, and the experimental data are in excellent agreement with the modified electron wave leakage model, which can be represented by Equation (1):

(1) $$Tc(d)=T_{C}\left(\infty \right)exp\left[\frac{-1}{N\left(0\right)V}\left(\frac{b}{d}+\frac{c}{d^{2}}\right)\right]$$

where Tc(∞) is the superconducting transition temperature at an infinitely thick film. b is the characteristic distance of electron waves leaking outside the superconductor, while c is a correction factor that describes the superconducting defects caused by the thin film thickness. N(0) is the density of states at the Fermi level, and V is the interaction potential. A decrease in film thickness significantly reduces the square resistance of the film, and the density of electronic states at the Fermi level and the superconducting transition temperature also decrease. In short, the research results on the superconducting properties of NbN crystals serve as a reference guide for the preparation of NbN-SNSPD.

**Figure 2:**
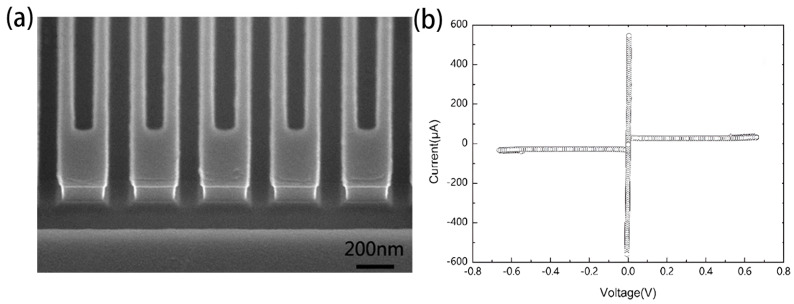
The morphology and electrical properties of NbN nanowire with d = 100 nm, width/spacing = 80 nm/80 nm. (**a**) SEM image of the side of the nanowire (**b**) I−V curve of superconducting NbN nanowire, which indicates that the device has good performance and may be used in the high-energy single photon detection experiment.

In order to meet various detection requirements, it is necessary to make more profound and sacrificial considerations for the preparation ofNbN thin films used in SNSPD. The thickness of SNSPD’s meandering nanowires often needs to be limited to within 10 nm (comparable dimensions to the size of a hotspot). Magnetron sputtering can be used to achieve the required film thickness by controlling the deposition rate and time. According to the superconducting properties, the nanowire thickness of the SNSPD affects its detection performance. Rall et al. [27] confirmed that the detection efficiency of SNSPD varies with the wire thickness and the wavelength. The absorption efficiency (ABS) of polarized photons parallel to the bending wire, which has relatively high detector absorption spectra in the visible and infrared regions, increases with the thickness of the wire. For the intrinsic detection efficiency (IDE), it shows the opposite thickness dependency relationship; that is, increasing the thickness will decrease IDE. These conclusions can be explained by the mechanism of hotspot and fluctuation-assisted detection [27]. The complex relationship should be taken into account in the process of optimizing SNSPD. In addition, the precise control of thickness is still limited by the film deposition technique. In particular, for some high-energy photons such as X-rays and gamma rays, thin films with a thickness of less than 10 nm exhibit very low absorption, with photon absorption rates of only 3.69% and 0.23% at 1 keV and 6 keV, respectively [28]. A high aspect ratio detector (with NbN thickness and meander linewidth close to 1:1) can achieve high ABS of X-ray photons and timing jitter below 10 ps by optimizing deposition and etching processes. Figure 2 shows the morphology and electrical properties of their NbN nanowire with a high aspect ratio.

**Figure 3:**
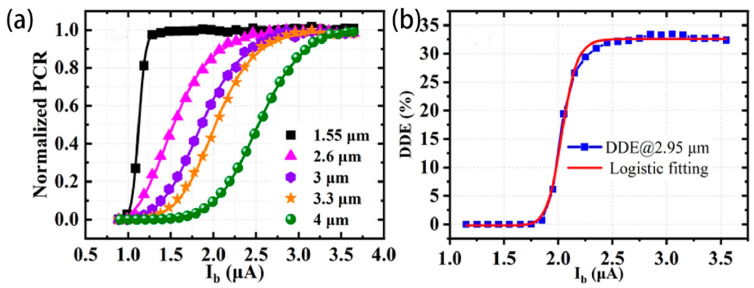
(**a**) Normalized PCR at various wavelengths as functions of the bias current (indicated by different colored symbols); (**b**) DDE as a function of the bias current at 2.95 µm wavelength. Copyright 2022, Optical Society of America.

NbNx with different stoichiometric ratios show various superconducting properties. However, the crucial question is whether they can perform well in detector applications. Under the hotspot mechanism, the relationship between the photon energy generated by a sufficient size of hotspots and the cross-sectional parameters of nanowires has been extensively studied [29]. The relationship between the cutoff wavelength and the film parameters can be represented by Equation (2):

(2) $$\begin{array}{c} {\lambda_{c}}^{-1}=\frac{3\sqrt{\pi }}{4hc\varsigma }\Delta^{2}wdN_{0}\sqrt{D\tau }\left(1-\frac{I_{B}}{I_{c}^{d}}\right) \end{array}$$

where λ*_c_* is the cutoff wavelength; λ*_c_* depends on the nanowire cross-section (i.e., thickness d of the film used for the device fabrication and width w of nanowire); superconducting energy gap Δ; quasi-particle diffusion coefficient *D*; density of electron states $N_{0}$; time constant of quasi-particle multiplication τ; quasi-particle multiplication efficiency ς; and ratio of the bias current $I_{B}$ to the departing critical current $I_{C}^{d}$. In order to improve the cutoff wavelength and extend the application of the detector to the infrared field, Henrich et al. [30] conducted a study that confirmed that changing the N content of a 4 nm thin film can also achieve a change in the cutoff wavelength. Decreasing the thickness of the film can potentially contribute to achieving this objective; however, it will still be subject to the various limitations mentioned above. They deposited NbN structures with different N contents by controlling the sputtering current I_sp_ (100~190 mA). Increasing the sputtering current (up to 190 mA) would enhance the metallic properties of NbN (increase in niobium content) and show a redshift (~25%) in the cutoff wavelength of the detector and a significant reduction in dark counts. These can be attributed to the increase in the residual resistivity ratio, electron diffusion coefficient, and resistivity value of the thin film [30]. Pan et al. [31] reported on the potential of γ-Nb_4_N_3_ thin films (with a lower N content compared to NbN) in the field of mid-infrared applications. They controlled the N/Nb film content at 0.63 and enhanced mid-infrared photon absorption by adding a gold reflector during the deposition process, achieving a saturated IDE at 4 µm and a device detection efficiency (DDE) of 32.5% at 2.95 µm, as shown in Figure 3; the device exhibits saturation plateaus for wavelengths up to 4 µm.

High-quality NbN film is crucial for ensuring the optimal performance of the detector. Some researchers improve the stability and quality of the film by optimizing the substrate material. It has been observed that the direct deposition of NbN on common silicon substrates leads to significant lattice mismatch, which in turn degrades the superconducting performance of the film [32,33]. Ilin et al. [32] found that there is an amorphous interfacial layer between the Si substrate and the NbN film. This phenomenon is caused by the lattice mismatch between Si and NbN, which results in atomic diffusion during the sputtering process. More concerning is the observation made by Semenov et al. [19] during their research on the optical properties of ultrathin NbN films deposited on sapphire. High-resolution transmission electron microscopy (HRTEM) and X-ray reflectometry showed that, regardless of the film’s thickness, an oxide layer would form on the surface of the film upon initial exposure to air. These issues can suppress the superconductivity of niobium nitride films, particularly in the case of nanoscale ultrathin films. Researchers select substrate materials with a lattice parameter close to that of NbN for the epitaxial growth of high-quality thin films. MgO has a very low lattice mismatch with NbN, making it an ideal substrate for the epitaxial growth of NbN films [34,35], thereby improving the uniformity and controllability of the film. Moreover, a buffer layer can be used between NbN and the substrate to reduce lattice mismatch and improve the optical performance of the detector. Bao et al. [36] used radiofrequency magnetron sputtering to adjust the sputtering parameters (400 W power, N_2_:Ar = 3:1, room temperature) to create the Nb_5_N_6_ buffer layers on the silicon substrate. They then evaluated the Nb_5_N_6_ buffer layers of various thicknesses under different sputtering pressures using Atomic Force Microscope (AFM). It was found that films with a buffer layer exhibited higher superconducting performance compared to those deposited directly on the substrate, and a 20 nm buffer layer could achieve a higher transition temperature ($T_{C}$ = 10.3 K) for a 3 nm film. Kobayashi et al. [37] epitaxially grew an AlN buffer layer on sapphire, which has a lattice match with the B-NbN plane (mismatch as small as −0.2%). The study showed that a 7 nm thick film with an AlN buffer layer has a good structure and superconducting performance, with a superconducting transition temperature of 11.3 K, which is sufficient for SNSPD applications.

The deficiencies of NbN also result in significant time jitter in the detector. In order to reduce defects and ensure uniformity in the thin film, researchers continuously optimize the sputtering parameters in search of optimal solutions. Merie et al. [38] focused on the effect of sputtering deposition temperature on the mechanical properties of niobium nitride films (hardness, adhesion). They used direct current magnetron sputtering to deposit three types of thin films on silicon substrates at different deposition temperatures (25 °C, 200 °C, and 400 °C). The mechanical structure of the films was observed using a scanning electron microscope (SEM). The experiments showed that an increase in deposition temperature leads to a decrease in film hardness and elasticity, as well as an increase in surface roughness, which affects the subsequent nanowire etching and uniformity. This phenomenon can be attributed to the polymorphic structure of NbN. Researchers have also investigated the adhesion and friction properties of NbN by varying the deposition time. They reached a similar conclusion that the deposition time also affects the microstructure of NbN crystals [39]. Najafi et al. [40] employed resistivity and superconducting transition temperature as two metrics to assess the quality and uniformity of the film. They optimized the heating conditions of the substrate and achieved the deposition of highly homogeneous films by utilizing a double-polished Si substrate and a SiN_x_ layer with a thickness of 300 nm. The detector demonstrated a low timing jitter of 24 ps and a high detection efficiency.

### 2.2. Atom Layer Deposition

Higher quality thin film, i.e., fewer defects, greater uniformity, and homogeneity, has always been a goal in the preparation of superconducting nanowires. ALD, with its self-limiting growth mechanism and atomic-level deposition, is widely used in the creation of high-quality thin films with precise thickness control [41,42].

**Figure 4:**
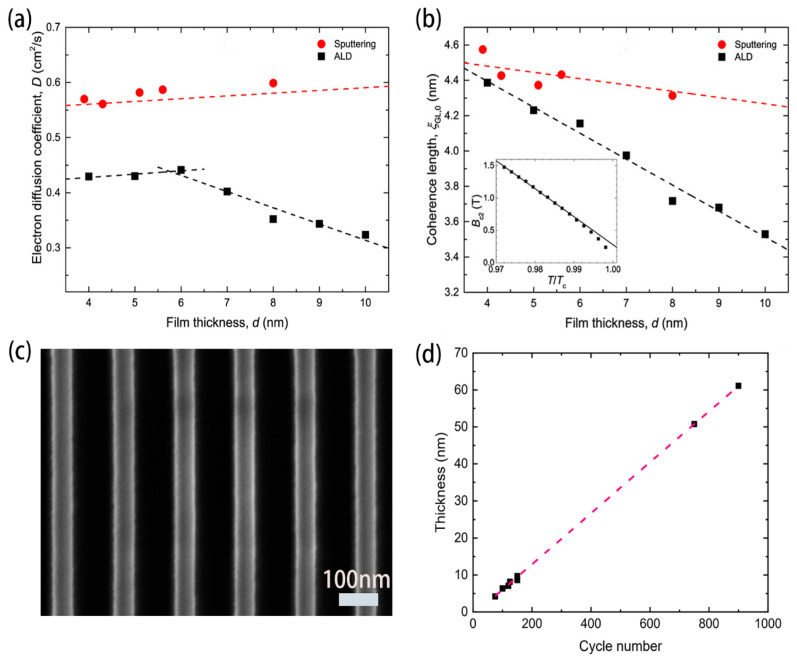
(**a**,**b**) Comparison of superconducting performance of films with different thicknesses under ALD and magnetron sputtering; (**a**) electronic diffusion coefficient D. Copyright 2019, IOP Publishing Ltd. (**b**) Superconducting coherence length. Copyright 2019, IOP Publishing Ltd. (**c**) Scanning electron microscope (SEM) image of the ALD/NbN nanowire with a linewidth (w) of 50 nm, showing exceptionally good uniformity. Scale bar, 100 nm. Copyright 2021, AIP Publishing. (**d**) The relationship between film thickness and the number of ALD cycles. Controlling the number of cycles allows for precise control of film thickness. Copyright 2021, AIP Publishing.

Knehr et al. [43] studied the suitability of the ALD thin film growth mechanism for SNSPD. During the deposition process, specific metal–organic compounds, tertbutylimido)-tris (diethylamino)-niobium (TBTDEN), were used as precursors and hydrogen plasma was used as an assistant. The deposition was set up in a large number of four-step cycles. They successfully developed SNSPDs made using ALD technology. As shown in Figure 4a,b, their conclusion was that the films by ALD had a smaller diffusion coefficient D and shorter coherence length ξ compared with magnetron sputtering. Linzen et al. [44] successfully prepared a 40 nm niobium nitride film using plasma-enhanced ALD (PEALD). By changing process parameters (plasma duration, substrate temperature, and hydrogen flow rate), NbN films with optimal superconducting performance were obtained. Cheng et al. [45] also used similar deposition materials and processes to prepare NbN films. Figure 4c shows that good control of thin-film deposition cycles resulted in a functional relationship between the number of deposition cycles and film thickness [45]. And the smooth film surface ensured the uniformity of the subsequent integration of long nanowires, as shown in Figure 4d. The final result achieved saturation of internal efficiency and low timing jitter in detectors operating at 1550 nm. Unfortunately, the native oxide layer on the film surface could not be avoided, and an oxygen peak was found between niobium NbN and SiN_x_ substrate in X-ray photoelectron spectroscopy (XPS), which also affected superconductivity [45].

Taylor et al. [46] prepared a large-area detector with infrared single-photon sensitivity (from 1310 nm to 2006 nm) using ALD, verifying the advantages of uniformity of ALD/NbN films, comparing with the larger roughness of columnar crystal films that could not be avoided during magnetron sputtering preparation. Knehr et al. [47] further demonstrated that ALD/NbN could maintain a high level of consistency in terms of thickness and roughness across the entire effective area of the wafer. However, the switching current density would undergo significant changes with the increase in the wafer size, possibly due to changes in the crystal structure.

In conclusion, ALD technology has unique advantages in realizing large-scale array detectors and producing uniform thin films. Precise control of film thickness is more convenient for research. So, it is an ideal technology platform for future SNSPD preparation.

### 2.3. Other Reported Deposition Techniques

**Figure 5:**
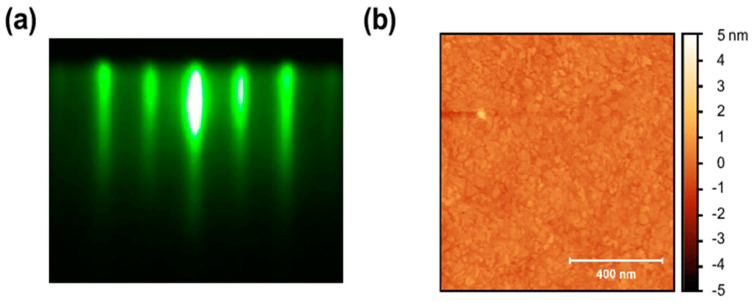
(**a**) RHEED showing the epitaxial film properties. The stripes in the pattern indicate a good two-dimensional growth mode of single crystal NbN. (**b**) Surface height map of NbN thin film under AFM. The root-mean-square roughness (Rrms) is less than 0.3 nm. Copyright 2020, AIP Publishing.

The deposition technique of chemical vapor deposition (CVD) in early work [48,49]. Nagai et al. [50] used a mixture of NbCl_5_, NH_3_, and H_2_ to deposit niobium nitride on Si and Al_2_O_3_ substrates. They found that the film structure and roughness of CVD were related to the growth temperature and deposition time. To obtain high quality ultra-thin films suitable for superconducting single-photon detection, Mercier et al. [51] conducted high-temperature CVD experiments in a two-chamber reactor. The first chamber uses chlorinated niobium wire to produce NbCl_x_, while the second chamber involves the chemical reaction between NbCl_x_ and NH_3_, with hydrogen serving as the carrier gas. They prepared niobium nitride with a high superconducting transition temperature of about 17 K and a higher tensile stress compared to magnetron sputtering. CVD has great potential in large-scale production and film thickness control. Another method in physical vapor deposition, molecular beam epitaxy (MBE), also has its advantages. Cheng et al. [52] reported the growth of a 7 nm thick niobium nitride film on AlN/sapphire substrates by radio frequency plasma-assisted MBE. As shown in Figure 5, the growth can be monitored using the reflection high-energy electron diffraction (RHEED) system. The NbN films exhibited a unique two-dimensional layer-by-layer growth mode and a relatively smooth surface (Rrms < 0.3 nm), as revealed by AFM. The etched nanowires showed a higher transition temperature ($T_{c}=12.1\;K)$ and a lower time response (τ = 5.4 ns) in the range of several nanometer-thick films. The AlN substrate platform has also been proven to be a very suitable choice. Wright et al. [53] demonstrated the influence of the MBE substrate temperature on the film structure and transition temperature. Compared with other techniques, MBE-deposited NbN films exhibit unique performance. For example, the highest transition temperature ($T_{C}=15.5\;K)$ was achieved at 1000 °C, resulting in films with a combination of two crystal structures. However, they encountered difficulties in forming high-quality crystalline films at substrate temperatures above 1300 °C. It is noticed that the single crystal structure of NbN at 800 °C had a lower transition temperature [53]. Pulsed laser deposition (PLD), an economical method, has also been attempted for the deposition of NbN films. Although it is mainly used for preparing ceramic materials like oxide films, its advantage lies in the high-quality film structure in terms of electronic and mechanical properties. In early work, Randolph et al. [54] used this method to grow high-quality NbN films on MgO substrates. They discovered that the film structure was influenced by the growth temperature and crystal orientation of the substrate. In subsequent work, the focus of film preparation shifted to electronic and mechanical properties to adapt to various applications. Farha’s team [55,56] studied the effects of laser parameters (energy, frequency), nitrogen partial pressure, and substrate temperature on the phase structure, hardness, and roughness of NbN films. Roch et al. [57] compared the superconducting properties of films deposited on four substrates and obtained the NbN film with a superconducting transition temperature of 15.5 K on C-Al_2_O_3_ substrates instead of MgO.

### 2.4. Preparation of NbTiN Thin Film

**Figure 6:**
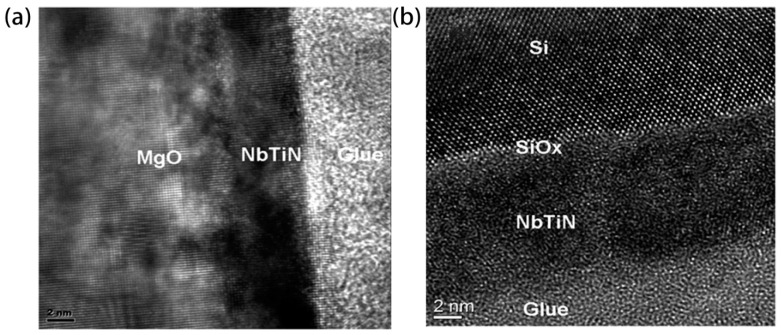
(**a**) Cross-sectional TEM imaging of a 5 nm thick NbTiN film on MgO substrate. (**b**) Cross-sectional TEM imaging of an 8 nm thick NbTiN film on Si substrate. Copyright 2019, Optical Society of America.

In addition to NbN, titanium niobium nitride (NbTiN) also stands out among superconducting crystal materials due to its excellent crystal structure. So far, the NbTiN materials used in superconducting single-photon detectors have all been prepared by reactive sputtering [58,59,60]. Similar to the preparation process of NbN, the target material is usually a niobium-titanium alloy, and discharge is carried out in a mixed gas of argon and nitrogen. And compared to NbN, the introduction of Ti is clearly the most crucial factor. Under the same preparation conditions, the dark count rate of NbTiN detectors is significantly lower than that of NbN detectors [61]. NbTiN thin films can achieve a smaller lattice mismatch with the substrate, resulting in improved film uniformity. Jia et al. [62] increased the Ti content to further reduce the lattice mismatch and film resistivity between the NbTiN film and MgO/Si substrate. As shown in Figure 6, both the interfaces between the film and substrate are quite clear and atomically sharp, and the NbTiN film perfectly extends the lattice structure of the MgO substrate, showing a crystalline state.

Chang et al. [63] utilized the flexibility of reactive co-sputtering deposition to prepare thin films with various values of x. Here, x is defined as the niobium-titanium metrological ratio. Excessive niobium content can lead to the formation of Cooper pairs that are difficult to break, thereby limiting the spectral response range and internal quantum efficiency. On the other hand, a lack of niobium content can result in a decrease in the material’s superconducting critical current, which can lead to significant time jitter in the detector. When x = 0.62, a 9 nm film covered an effective detection area of 20 μm and showed optimal temporal performance.

In addition, the kinetic inductance of NbTiN can be smaller in subsequent nanowire fabrication (~25% lower), which also reduces the timing jitter of the detector [58]. Due to the smaller resistivity and higher sensitivity of NbTiN, NbTiN-SNSPD shows a greater potential in the field of infrared detection [64]. Similarly, achieving purity, uniformity, and defect control in NbTiN films presents significant challenges during the fabrication process. Makise et al. [59] summarized the effects of nitrogen partial pressure and substrate material on the crystal structure and superconducting performance of NbTiN in magnetron sputtering. An increase in nitrogen concentration ratio under different substrate materials showed an increase in lattice constant and a decrease in deposition rate. Steinhauer et al. [65] demonstrated the universality of NbTiN deposited by reactive co-sputtering at room temperature in waveguide-integrated detectors (SSPDs). NbTiN can achieve good superconducting and optical properties on various substrates under universal sputtering formulations.

Similar to NbN, the thickness of the NbTiN film needs to be comparable to the coherence length of the superconductor. The impact of the film’s thickness on the performance of the detector is undoubtedly significant. Changes in thickness can lead to a series of interconnected changes in superconducting performance, including critical temperature, critical current, cutoff wavelength, light absorption efficiency, and internal quantum efficiency. The choice of thickness needs to be carefully considered. Zadeh et al. [66] focused on improving the overall performance of the detector for the 1550 nm telecommunications wavelength. Although thin films with a thickness of more than 10 nm exhibit good light absorption efficiency and a large critical current under FDTD simulation, the saturation level of IDE is not high at this time. On the other hand, the thickness of the thin film that is too low is limited primarily by processing technology. Additionally, it will result in a decrease in critical current. They selected a thin film with a thickness of 8.4 nm and made continuous improvements to the geometric structure of the subsequent nanowires, resulting in favorable outcomes. Secondly, attention should also be paid to the surface oxide layer of materials like NbN. After being exposed to air, the Ti and Nb on the surface of the NbTiN film will oxidize. In a 4 nm thick film, an oxide layer with a saturation value of approximately 1.5 nm may form, resulting in a decrease in surface quality [67]. The solution can be to incorporate suitable optical cavity structures during the preparation process or decrease the thickness of the oxide layer through subsequent thermal treatment of the film.

Table 2 summarizes the superconductivity properties of thin films under various deposition techniques and process parameters.

## 3. Preparation of NbN Nanowires

As we know, the preparation of NbN thin films is only the preliminary step in the fabrication of SNSPD. A complete processing step normally includes thin film preparation, electrode processing, nanowire geometric pattern etching, and optical structure processing. After preparing the thin film and electrodes, the required nanowire structures need to be drawn on them. The most common nanowire structure reported is drawn as a meandering curve with a width in the nanoscale range and a length in the micrometer range in order to achieve critical response and superconducting-normal switching. From the initial nanowire drawn by Gol’tsman et al. [6] to the optimization of various graphical definitions and geometric structures, significant improvements in performance and application requirements have been achieved. In this chapter, the preparation and optimization techniques for nanowire etching from the deposited NbN thin film are reviewed and analyzed.

### 3.1. Geometric Pattern Definition

The patterning of NbN/SNSPD nanowires is typically achieved through electron beam lithography (EBL), followed by reactive ion etching (RIE) to transfer the pattern onto the NbN film. SF_6_ [72] or CF_4_ [17] are ideal etching agents for EBL patterning. Other factors to consider in the process include the selection of EBL resist, the proximity effect of the EBL, etching time, and line uniformity, all of which affect the optimization of the etching process and, consequently, affect detector performance.

The uniformity and consistency of nanowires play a crucial role in achieving detectors with high yield and optimal performance. The non-uniformity of nanowires can be attributed, in part, to the etching processes employed and the choice of materials. Polymethyl methacrylate (PMMA) material is widely used as the EBL resist due to its excellent transparency and high processing flexibility [34,72]. PMMA is widely used in etching processes. Zhang et al. [34] used a 60 nm PMMA layer as an etch-resistant mask on a few-nanometer-thick film to pattern nanowires that were 50 nm wide. The thicker PMMA layer also serves as a protective barrier for the film during etching. It was found that the uniformity and edge roughness of the nanowires significantly affect the detector’s critical current, which in turn affects the timing jitter and dark count rate. Charaev et al. [73] considered the impact of line width. They compared positive and negative PMMA resists for patterning under the same etching conditions and found that the negative PMMA resist is more stable. It can improve resolution by reducing its thickness, allowing for a smaller nanowire width (w) to be achieved. This significantly increases the critical current density (~30%) and cooling efficiency, resulting in a 20% increase in the operated current. Negative HSQ resistance can also improve the resolution of etching, which has more potential in achieving optimized patterns, despite its poor adhesion to the NbN film. Stern et al. [74] used HSQ as an etch-resistant layer prior to NbN etching and observed an improvement in nanowire uniformity based on SEM images. Najafi et al. [40] applied a dip of tetra-methyl-ammonium hydroxide (TMAH) to the NbN films for a maximum of 15 seconds, which minimizes damage to the film while ensuring that HSQ adhesion is not significantly sacrificed. MENG et al. [75] replaced PMMA with a fractal nanowire structure using HSQ lithography to partially eliminate adhesion defects that may arise with HSQ.

The proximity effect of electron beam lithography significantly affects the uniformity and resolution of the pattern, particularly in nanoscale etching. Zadeh et al. [72] studied the effect of nanowire cross-sectional size on timing jitter in detectors. Local defects in ultra-thin film materials, such as minor changes in line width, can lead to significant timing jitter and noise. The factors that cause variations in line width include the tone of electron beam lithography, the thickness of the resist layer, and the choice of substrate.

In the face of the significant unpredictability and the limitations of resolution in electron beam lithography, researchers have explored alternative methods, including local oxidation lithography with AFM [76] and nonlinear femtosecond optical lithography [77], which all aim at improving the resolution. In addition to modifying the nanowire fabrication process and etching conditions, the performance of SNSPDs can also be enhanced through a post-processing method. Zhang et al. [14] utilized helium ion irradiation to alter the physical structure and superconducting properties of thin films, taking advantage of NbN’s excellent radiation resistance and a high tolerance for defects. This method can significantly improve the IDE of SNSPDs while ensuring high system detection efficiency.

### 3.2. Nanowire Size and Geometrical Parameters

The size and geometrical parameters of NbN nanowires we describe include the topological structure, the active area, line width, spacing, and filling factor (f). These designs and process parameters greatly affect the overall characteristics of the detector, such as IDE, count rate (CR), DCR, and timing jitter. Currently, although SNSPDs integrated with optical fibers can improve the coupling efficiency through optical fiber alignment sleeves [7,14] and integrated optical cavity structures [78,79], optimizing the effective photosensitive area of the nanowire is a direct method for improving IDE. Yang et al. [80] used a high-resolution EBL process to increase the f (from 0.4 to 0.9) to increase absorption, combined with an optical cavity structure to achieve high coupling efficiency. However, it was found that under high f, detection efficiency DE did increase but did not exceed expectations (the highest device DE value of 20% was on the f of 0.6). Even if the critical current density of SNSPDs were suppressed (decreased by 35% from the f of 0.4 to 0.9), there would have an impact on the cutoff wavelength and bandwidth of SNSPDs. Li et al. [81] addressed this issue by developing an asymmetric nanocavity structure made of metal–insulator–metal. This structure uses an absorbing cavity and still maintains high-efficiency coupling with a nanowire f of 0.2, reducing the response time (~40%). In addition, since the introduction of SNSPD, there has been a proposal to expand the sensitivity range of the detector by reducing the linewidth. Marsili et al. [82] reported that the NbN detectors they manufactured exhibited limited sensitivity above 50 nm, with negligible detection efficiency observed at shorter wavelengths. The sensitivity of SNSPD below a linewidth of 50 nm can be extended to a wavelength of 5 μm, below a linewidth of 50 nm. Under a linewidth of 30 nm, the detectors show good detection efficiency (2.6~5.5%) over a wavelength range of 0.5 μm to 5 µm.

**Figure 7:**
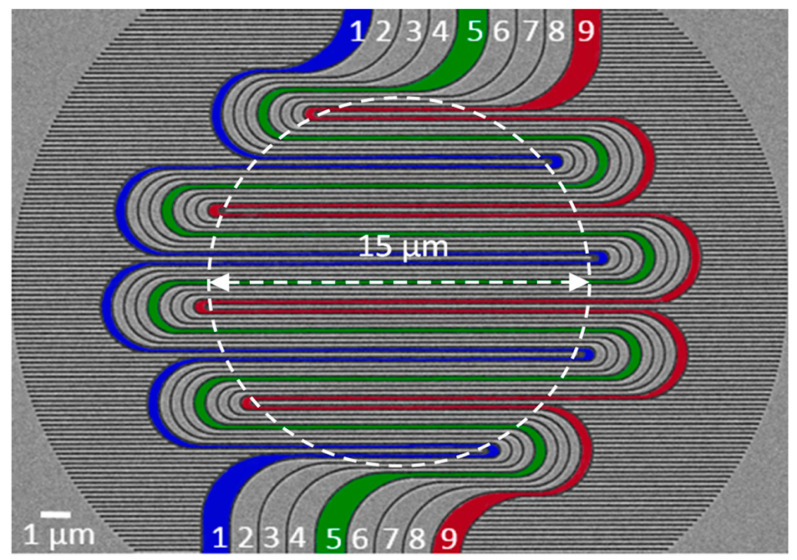
SEM image of a nine interleaved nanowire SNSPD array (active area of 15 μm in diameter as denoted by the dashed circle). Numbers 1 to 9 represent nine compact nanowires. Pseudo-colors in blue, green, and red are used to indicate three out of the nine nanowires. Copyright 2018, AIP Publishing.

Increasing the active area of nanowires to achieve SNSPDs’ large area detection while increasing their count rate and detection efficiency is a major research direction. However, common methods for achieving large-area nanowire arrays, such as extending a single nanowire, increase the kinetic inductance of the SNSPD, which results in higher timing jitter and longer recovery time [83,84]. Moreover, the difficulty of fabrication and product uniformity can also affect detection. A feasible solution is to create multiple NbN nanowires. Huang et al. [85] demonstrated a structure composed of nine interleaved NbN nanowires with an effective area of 15 μm in diameter, as shown in Figure 7. The length of each nanowire was reduced compared to common nanowires, which reduced the kinetic inductance. Through further work, the system detection efficiency of this structure was improved to over 90%.

**Figure 8:**
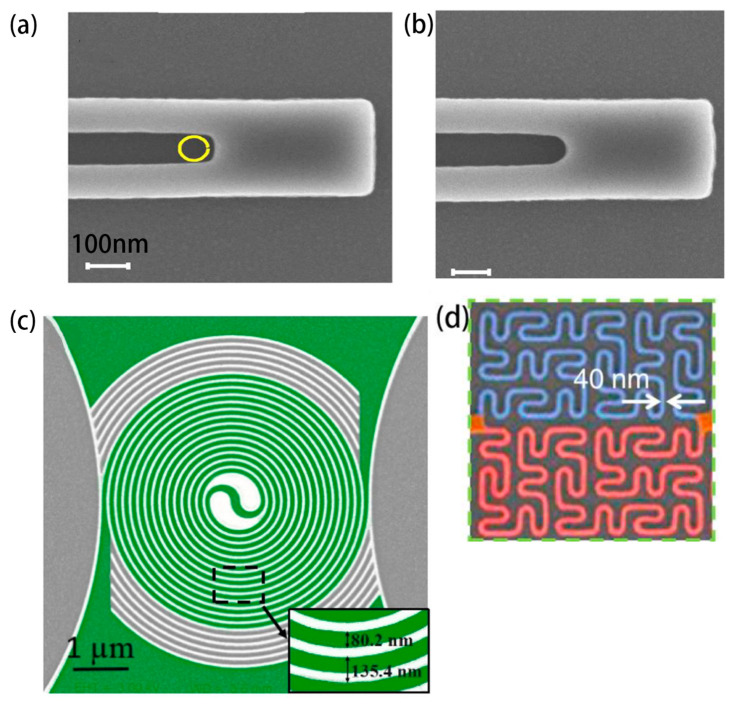
Different geometric structures of nanowires. (**a**) Common meandering nanowire structure with sharp bend; (**b**) Serpentine structure with rounded bends, which reduces the effect of current crowding and dark counts compared to the former. (**a**,**b**) share the same length scale (100 nm). Copyright 2012, Optical Society of America. (**c**) Spiral structure, which reduces both current crowding and polarization dependence of detection. Copyright 2017, IOP Publishing Ltd. (**d**) Fractal structure, which reduces polarization dependence of detection. Copyright 2020, Optical Society of America.

One thing to note is the current crowding effect in nanowire structures. So far, studies on meandering nanowires have been the most extensive. The current density in the bending area of a typical meandering nanowire structure needs to be carefully considered. This is because the current density inside the bend is usually higher than on the outside of the bend, which restricts the critical current of SNSPD [86,87]. The structure of nine staggered nanowires, as reported in reference [85], reduces the current crowding effect by rounding off. Akhlaghi et al. [88] designed nanowires with different bend shapes that were 100 nm wide, as shown in Figure 8a,b. The nanowires, which were curved in a circular shape, eliminated the current crowding effect while significantly reducing dark counts. Xiong et al. [89] also avoided this effect by reducing the proportion of serpentine structures while thickening the curved parts while maintaining a high filling factor. As it stands, some researchers have abandoned meandering structures and instead design spiral structures. This approach solves the issue of the current crowding effect and also reduces the polarization response of the detector (the detection efficiency is dependent on the polarization degree of incident photons). Henrich et al. [90] prepared spiral nitride nanowires on sapphire. For a 300 nm line width, the critical current of the spiral nanowire structure increased by 20% compared to the serpentine structure. The critical current of nanowires with a line width of 100 nm was not greatly increased, but their uniform spiral structure significantly increased the detection efficiency. The detection efficiency was increased by 1.5 times in the visible photon wavelength range and 2.7 times in the infrared range. Huang et al. [91] also verified the characteristic of the spiral structure in eliminating the current crowding effect, even at high f. Their SNSPDs had a detection efficiency of 52.5% at a working wavelength of 1550 nm while also reducing the detector’s dependence on photon polarization. Recently, research on fractal structures that eliminate polarization dependence has also been reported [75,92].

### 3.3. Microwire Detector

**Figure 9:**
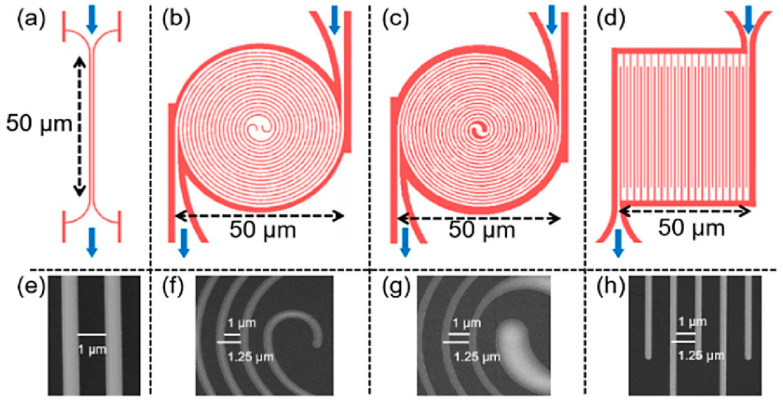
Four different structure diagrams and SEM magnified images of SMSPDs. The blue arrow indicates the direction of the electric current. (**b**,**f**) are improved double-helix structures, which optimize the angular curvature and geometry of the center compared to the regular double spiral strip (**c**,**g**). (**a**,**e**), the short micrometer bridge; (**d**,**h**), the conventional meandered strip. Copyright 2021, Optical Society of America.

Because of the “hotspot” mechanism, the line width of the superconducting nanowire is primarily in the nanometer range (ranging from 50 nm to 300 nm). To improve the detection efficiency and sensitivity, it is desirable to prepare nanowires with a width comparable to the size of the “hotspot”. In the past, this model was primarily used for qualitative research. However, in recent years, new theories have pointed out that when the initial current density is uniform and close to the critical current density, the photon detection efficiency is not necessarily related to the geometric size of the nanowire, such as the line width [93]. Korneeva et al. [94] were the first to experimentally verify this theory. The micrometer-scale NbN bridge they prepared can achieve single-photon detection. This microwire detector can be called SMSPD. Compared to SNSPD, the process requirements for SMSPD can be reduced, making it more economical. Xu et al. [95] also prepared the NbN micrometer structure, as shown in Figure 9. They believe that a high filling factor is needed for the microwire detector to perform well. The optimized spiral microwire structure is shown in Figure 5b, and the highest detection efficiency can approach saturation (~92.2%), and the f is 0.8 [95]. In summary, research on SMSPD is still in its early stages, and more effort needs to be invested in mechanism research, process optimization, and overall performance improvement. Table 3 shows the performance of detectors fabricated with nanowires or microwires using various preparation processes.

## 4. Outlook

Superconducting NbN has already established a well-developed fabrication process, and the utilization of SNSPDs has demonstrated significant promise for various applications. However, there are still unresolved matters that require immediate attention:

(1) Comprehensive Performance: SNSPD has multiple performance indicators and parameters. Optimizing a single performance is insufficient to meet current user demands. SNSPDs designed for Time-Frequency Quantum Key Distribution (TF-QKD) applications must balance high detection efficiency with low DCR. In the fields of satellite mapping and laser ranging, SNSPD has high requirements for SDE, DCR, and timing jitter. Some special applications require extending the detection wavelength to the mid-infrared or achieving low photon polarization dependence. The improvement of two or more indicators should be emphasized in future research endeavors. The connection and contradiction between SDE and temporal characteristics, as well as the relationship between manufacturing processes and product quality, pose significant challenges. One potential solution is to explore the use of superconducting materials that combine Nb-based crystalline materials with amorphous materials like WSI. This approach could potentially leverage the advantages of both materials to achieve improved overall performance. By utilizing various thin film manufacturing processes and continuously optimizing process parameters, higher-quality thin films and nanowires can be achieved. Additionally, the design of precise and targeted optical cavity structures can also be considered. Another issue worth discussing is whether it is possible to define standards for comprehensive performance indicators within a specific application to assess various SNSPDs. This is a topic that may need to be discussed in the future.
(2) Practicality: Various factors, including system size, weight, power consumption, operability, and cost, will collectively influence the practicality of the system. In certain applications, such as those involving the installation of a detector on a satellite or other spacecraft, it is crucial to impose strict limitations on the SWAP (size, weight, and power) of the SNSPD system. Therefore, it is imperative to allocate more resources towards conducting further research on the advancement of lightweight and compact refrigeration systems. And the future success of the product will depend on its ability to provide simplified operability and affordable prices, as these factors are crucial in attracting a broader user base. The strategy for accomplishing this objective could involve raising the working temperature. For instance, one approach to address this issue is to search for superconductors with higher superconducting transition temperatures to reduce strict low-temperature requirements. Xing et al. [101] made attempts, and their Yttrium Barium Copper Oxide (YBCO) microwire detector can achieve a critical temperature as high as 89 K and photon response at 85 K. However, YBCO superconductors face challenges in achieving a wide spectral response, particularly in terms of insensitivity to low-energy photons. This limitation results in low detection efficiency and makes it difficult to apply them in SNSPDs. Another approach involves the simplification of optical coupling and the integration of diverse optical and mechanical components in order to facilitate operation and enable adjustable functionalities.

## Figures and Tables

**Table 1:** Lattice structure and superconducting transition temperature of Nb-group nitrides.

NbN Phase	Crystal Structure	$\mathrm{Reference}\;\mathrm{Transition}\;\mathrm{Temperature}\;T_{C}$ (K)
β-Nb_2_N	Hex.	<9.5
γ-Nb_4_N_3_	Tetr.	12.2–7.8
δ-NbN	Cub.B1	7.2
δ’-NbN	Tetr.	16.5–9.7
ε-NbN	Hex.	<1.20
Nb_5_N_6_	Hex.	<1.77
Nb_4_N_5_	Tetr.	8.0–8.5

**Table 2:** Superconducting performance of thin films under different sputtering conditions.

Material	Substrate	Process	T_s_ (°C)	d (nm)	T_C_ (K)	ρ ^1^ (μΩ cm)	Ref.
NbN	SiO_2_	sputtering	ambient	5	7.8	253	[68]
NbN	HfO_2_/SiO_2_/Si	sputtering	300	28	13	-	[20]
NbN	R-Al_2_O_3_	sputtering	750	5.3	11.54	-	[19]
NbN	MgO	sputtering	-	3.3~5	11.5	200/d = 5 nm	[26]
NbN	Si	sputtering	825	10	12	-	[32]
NbN	SiO_2_/Si	ALD	300	<10	~8	240	[45]
NbN	R-AL_2_O_3_	ALD	350	6	11.3	300	[43]
NbN	Glass	ALD	350	10	12.1	325	[44]
NbN	Al_2_O_3_	HTCVD	1300	49	17.06	62.8/17.25 K	[69]
NbN	AlN/Al_2_O_3_	MOPVD	850	7	11	-	[37]
NbN	AlN/Al_2_O_3_	MBE	1100	7	12.1	100	[52]
NbN	C-Al_2_O_3_	PLD	600	50	15.5	104	[57]
Nb_1−x_Ti_x_N ^2^	Al_2_O_3_	HTCVD	1100	10	13.1	167/20 K	[70]
Nb_1−x_Ti_x_N ^3^	SiO_2_	Sputtering	-	9	11.26	-	[63]
NbTiN	SiO_2_/Si	Sputtering	-	5	8.4	156.3/20 K	[71]

^1^: Data without further explanation are measured at room temperature. ^2^: x represents different Nb/Ti ratios, with the highest transition temperature (13.1 K) and a resistance of 167 Ω (at 50 K) obtained when x = 0.46. ^3^: Here, x = 0.62.

**Table 3:** Performance of SNSPDs with different NbN nanowire structures.

Substrate	d (nm) Geometry		w (nm)	^1^ A (µm)	SDE/λ (nm)	DCR (Hz)	Jitter (ps)	Ref.
Si (DBR)	7	Meandering (array)	100	^2^ R = 300	50%/1064	30	-	[96]
Si (DBR)	6.5	Meandering	160	R = 100	65%/532	100	82	[17]
SiO_2_/Si	6.5	Meandering	80	18 × 18	73.6%/1550	0.1	-	[97]
SiO_2_/Si	5	Meandering	80	R = 14	76%/1550	10^4^	~70	[98]
Si (DBR)	~8	Meandering	81	18 × 18	92%/1550	-	-	[99]
SiO_2_/Si	6	Meandering	60	R = 15	63%/2000	2	-	[100]
Al_2_O_3_	4	Spiral	100	R_1_ = 4.2 R_2_ = 0.6	27.6%/400~600	-	-	[90]
SiO_2_/Si/SiO_2_	6.5	Spiral	80	R = 16	52.5%/1550	100	-	[91]
SiO_2_/Si	6	9 interleaved	90	R = 15	>50%/1550	200	-	[85]
Si (DBR)	7	Spiral (SMSPD)	1000	R = 50	92.2%/1550	200	50	[95]
SiO_2_/Si	9	Fractal	40	8.6 × 8.6	60%/1550	220	45	[75]

^1^: A represents the active area of the nanowire. ^2^: R is the diameter.

## Data Availability

The data presented in this study are available on request from the corresponding author.
